# *Miscanthus sinensis* contributes to the survival of *Pinus densiflora* seedlings at a mining site via providing a possible functional endophyte and maintaining symbiotic relationship between *P. densiflora* and endophytes from high soil temperature stress

**DOI:** 10.1371/journal.pone.0286203

**Published:** 2023-05-23

**Authors:** Toshikatsu Haruma, Kohei Doyama, Xingyan Lu, Kenta Noji, Hayato Masuya, Takahiko Arima, Shingo Tomiyama, Keiko Yamaji

**Affiliations:** 1 Faculty of Engineering, Division of Sustainable Resources Engineering, Hokkaido University, Kita, Nishi, Kita-ku, Sapporo, Hokkaido, Japan; 2 Graduate School of Life and Environmental Sciences, University of Tsukuba, Tennoudai, Tsukuba, Ibaraki, Japan; 3 Department of Mushroom Science and Forest Microbiology, Forestry and Forest Products Research Institute, Tsukuba, Ibaraki, Japan; Universiti Pendidikan Sultan Idris, MALAYSIA

## Abstract

At a sedimentary site in an old mine site, *Miscanthus sinensis* formed patches, where *Pinus densiflora* seedlings could grow better compared with those outside the patches, indicating that *M*. *sinensis* would improve *P*. *densiflora* seedling establishment. The purpose of this study was to understand the mechanisms by which *M*. *sinensis* facilitates the survival of *P*. *densiflora* seedlings by considering the soil properties, heavy metal tolerance, and root endophytes in *P*. *densiflora* seedlings at the sedimentary site. The sedimentary site, which is a bare ground, contained high concentrations of Fe, indicating that plants should be exposed to Fe and high soil temperature stresses. Measurement of soil temperature revealed that *M*. *sinensis* suppressed sharp increases and alternation of soil temperature, resulting in reducing high soil temperature stress in *P*. *densiflora* seedlings. To adapt to the Fe stress environment, *P*. *densiflora* outside and inside the patches produced Fe detoxicants, including catechin, condensed tannin, and malic acid. *Ceratobasidium bicorne* and *Aquapteridospora sp*. were commonly isolated from *P*. *densiflora* seedlings outside and inside the patches as root endophytes, which might enhance Fe tolerance in the seedlings. *Aquapteridospora* sp., which is considered as a dark-septate endophyte (DSE), was also isolated from the roots of *M*. *sinensis*, suggesting that *M*. *sinensis* might play a source of a root endophyte to *P*. *densiflora* seedlings. *Ceratobasidium bicorne* could be classified into root endophytes showing symbiosis and weak pathogenicity to host plants. Therefore, high soil temperature stress would weaken *P*. *densiflora* seedlings, causing root endophytic *C*. *bicorne* to appear pathogenic. We suggested that *P*. *densiflora* could adapt to the Fe stress environment via producing Fe detoxicants, and *M*. *sinensis* would facilitate the establishment of *P*. *densiflora* seedlings in the sedimentary site by providing a DSE, *Aquapteridospora* sp., and maintaining symbiosis of *C*. *bicorne* from high soil temperature stress.

## Introduction

The heavy metal environments in the world originate from anthropogenic activities such as mining, whereas some occur naturally, such as serpentinite sites. There are numerous old mine sites in Japan, and acidic mine wastewater containing high concentrations of harmful metals is a significant problem [1]. To ameliorate this problem, heavy metals can be removed from wastewater as sludge and stored at sedimentary sites. Phytostabilization is a relatively inexpensive and widely adaptable method for preventing the diffusion of sediments containing high concentrations of heavy metals [2,3]. High concentrations of heavy metals including Al are fatal to plants without heavy metal tolerance because of metal toxicity, such as generation of reactive oxygen species and enzyme inactivation [4–9]. Therefore, heavy metals can disturb vegetation by creating a toxic effect and hampering plant growth [10,11]. In contrast, natural vegetation has been observed in heavy metal environments [12–14], indicating that plants naturally growing in heavy metal environments have adapted to heavy metal environments [15–17]. Elucidation of heavy metal tolerance in native plants in sedimentary sites would be useful for the revegetation of sedimentary sites using heavy metal-tolerant native plants.

At sedimentary sites, it is important to introduce pioneer plants with tolerance to toxic metals. *Miscanthus sinensis*, which can be observed in various old mine sites as a pioneer species [18], has Al tolerance mechanisms [19,20]. Harmful metal tolerance in plants can be explained by [17,21–24]: 1) inhibition of heavy metal invasion into cells by adsorption to cell walls, 2) reduction of heavy metal permeability through the cell membrane, 3) production of polypeptides including sulfur, 4) sequestration of heavy metals chelated by organic acids and phenolic compounds into vacuoles, 5) active removal of heavy metals from the cell, and 6) removal of reactive oxygen species generated by harmful metals. Root endophytes and symbiotic microbes growing in roots harmful metals tolerance of plants [25–29]. In our previous study [20], *M*. *sinensis* suppressed the transfer of Al to aboveground parts and produced chlorogenic acid to detoxify Al in roots. Additionally, root endophytes can enhance Al tolerance in *M*. *sinensis*. Therefore, it is important to clarify the tolerance of plants to harmful metals by considering their interactions with the root endophytes.

At the sedimentary site, it was observed that *M*. *sinensis* naturally grew and formed patches, and *Pinus densiflora* seedlings grew well inside these patches compared with those outside the patches. We hypothesized that *M*. *sinensis* possessing harmful metal tolerance could enhance the growth of *P*. *densiflora* seedlings at the sedimentary site. The purpose of this study was to clarify the mechanisms by which *M*. *sinensis* enhances *P*. *densiflora* establishment in sedimentary sites by considering harmful metals tolerance in *M*. *sinensis* and *P*. *densiflora* seedlings. We considered the effect of *M*. *sinensis* on three main factors: 1) soil properties, including harmful metal concentrations, water content, and soil temperature; 2) heavy metal tolerance in *P*. *densiflora* such as suppression of harmful metal uptake, enhancement of nutrient element uptake, and harmful metal detoxicants; and 3) infection rate and species of microbes (endophytes and ectomycorrhiza) growing in roots. Through these experiments, we discussed whether *M*. *sinensis* could contribute to the survival rate of *P*. *densiflora* and could enhance vegetation succession.

## Materials and methods

### Survival rate and mortality factor of *P*. *densiflora* seedlings outside and inside the patches

**Fig 1:**
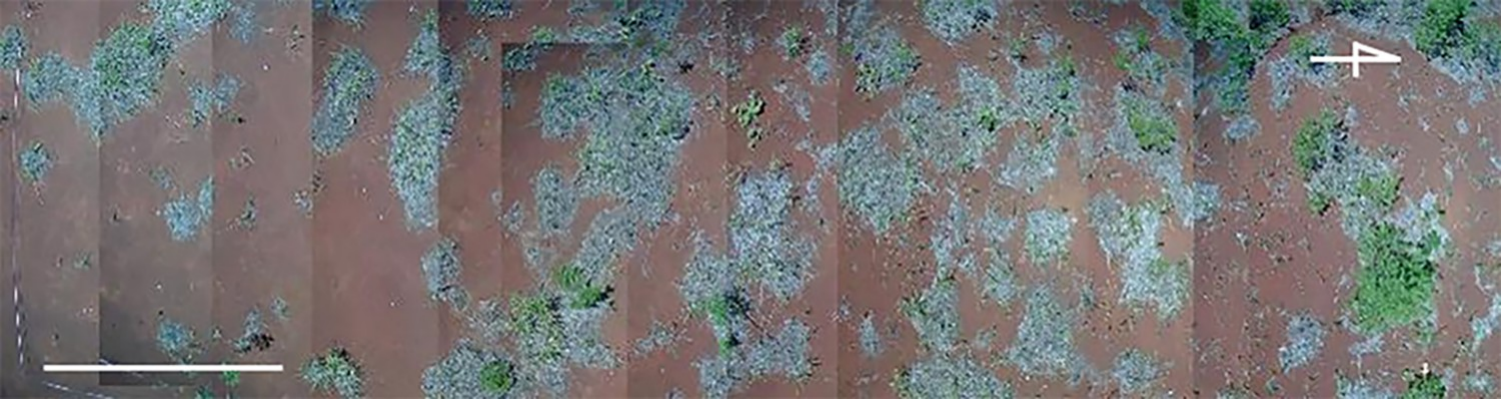
Plot for observation of survival rate of *Pinus densiflora*. The overview of the plot taken in July 2021; scale bar indicates 5 m. The pale green color shows patches of *Miscanthus sinensis*. The brown color shows soil surface without vegetation.

Our study site was a sedimentary site located in an old mine in Akita Prefecture, Japan. We obtained permits and approvals for the work from a company which has the mine site. Any protected species were not sampled. At the site, lime and harmful metals present in the acid mine wastewater were precipitated, and the soil was classified as man-made soils according to the FAO-UNESCO system [30]. At the study site, *M*. *sinensis* formed patches (Fig 1), and current-year *P*. *densiflora* seedlings grew naturally outside and inside these patches. To observe the survival rate of *P*. *densiflora* seedlings, we established a plot (north–south: 35 m; east–west: 10 m) (Fig 1) at the site. A total of 51 patches of *M*. *sinensis* were observed in the plot. A total of 124 and 77 current-year *P*. *densiflora* seedlings were observed outside and inside 42 patches, respectively, as identified using numbering tapes in September 2019. Survival rates were observed in July and September 2020–2022. Dead seedlings were collected to identify the mortality factors (disease, physical damage, and lost) using a stereo microscope (VMF2x, Olympus, Tokyo, Japan). The main symptom was root rot. After observations, possible pathogenic fungi were isolated from the roots of *P*. *densiflora* seedlings, whose main mortality factor was disease. The roots were surface-sterilized with 70% ethanol followed by 1% sodium hypochlorite solution and again with 70% ethanol each for 1 min. They were then rinsed twice with sterile deionized water for 5 min to remove the reagents and were dried on sterile filter paper on a clean bench for 5 min. The surface-sterilized roots were cut into approximately 10-mm segments with a sterile scalpel, placed on 1% malt extract agar medium, and incubated at 23°C in the dark for 2 weeks.

Fungal detection percentages (%) were calculated by means of the following formula:

$$Root\;endophyte\;detection\;rate\;(\%)=\frac{N_{d}}{N_{t}}\times 100$$ (1)

where N_d_ is the number of dead seedlings from which possible pathogenic fungi appear, and N_t_ is the total number of dead seedlings used for isolation.

### Harmful metal concentrations, pH (H_2_O) water content and temperature in soil outside and inside patches

In September 2019, soil samples (200 × 200 × 200 mm volume) outside and inside the patches were collected from randomly selected four patches. After air-drying at 20°C for 1 week, the soil was passed through a sieve (< 2 mm). Soil properties, such as pH (H_2_O), concentrations of harmful metals (Al, Fe, Cu, Mn, and Zn), and available Fe were measured. The pH (H_2_O) was determined using a pH meter (F-22, HORIBA, Kyoto, Japan). The harmful metals were quantified using inductively coupled plasma-optical emission spectrometry (ICP-OES; Agilent 720, Agilent Technologies, Santa Clara, CA, USA), after digestion in concentrated HNO_3_–HClO_4_ (1:4 v/v) at 140°C. Available Fe was extracted according to a modified method [31] below: 8 g of air-dried soil was added to 20 mL of 1.2 mol/L sodium acetate solution (pH 4.8). Available Fe was extracted by shaking at 100 rpm for 1 h, and its concentration was measured via ICP-OES. For all measurements, the results of four replications were averaged, and standard errors (SEs) were calculated.

In July and September 2020–2022, water content in 20 soil samples outside and inside the patches was measured using a soil moisture meter (HH2; Daiki Rika Kogyo Co., Ltd, Konosu, Japan). From July to September 2022, soil temperature at 5 cm depth was measured every 1 h outside and inside the two patches using a soil thermometer (TR-52i, T & D Co., Matsumoto, Japan).

### Elemental concentrations in *M*. *sinensis* and *P*. *densiflora* seedlings growing outside and inside the patches

Five individuals of *M*. *sinensis* growing at our study site were collected in July 2020. The collected samples were washed with deionized water to remove soil particles [32,33] and separated into aboveground parts, dead leaves, rhizomes, roots, and root skins. The separated tissues were dried at 80°C for 48 h, ground, and pyrolyzed in concentrated HNO_3_ at 130°C. The concentrations of harmful metals (Al, Fe, Cu, Mn, and Zn) and nutrient elements (Ca, K, Mg, and P) in the plant tissues were measured using ICP-OES. The concentrations of harmful metals in each tissue of the five *M*. *sinensis* samples were averaged, and SEs were calculated. The transfer factors of harmful metals (ratios of plant tissue concentrations to soil concentration) were calculated according to [34] as follows:

$$Transfer\;factor=\frac{Harmful\;metal\;concentration\;in\;plant\;tissues\;(mg/kg)}{Harmful\;metal\;concentration\;in\;soils\;(mg/kg)}$$ (2)

The results of the five replications were averaged, and SEs were calculated.

In July and September 2020, eight current-year seedlings of *P*. *densiflora* were collected from outside and inside the patches, respectively. In July and September 2021, eight one-year seedlings of *P*. *densiflora* were collected from the outside and inside the patches, respectively. The collected samples were washed with deionized water to remove soil particles [32,33] and separated into aboveground parts, hypocotyls, and roots. The separated tissues were dried at 80°C for 48 h, ground, and pyrolyzed in concentrated HNO_3_ at 130°C. The concentrations of harmful metals and nutrient elements in the plant tissues were measured using ICP-OES. The results of the eight replications were averaged, and the SEs were calculated.

### Analysis of phenolic compounds and organic acids in roots of *M*. *sinensis* and *P*. *densiflora* seedlings

Roots of *M*. *sinensis* used for elemental analysis mentioned above were also employed for phenolic compounds analysis. The roots of the five individuals of *M*. *sinensis* were washed with deionized water. The roots were cut into pieces with scissors in methanol for the extraction of phenolic compounds for 5 days at 23°C in the dark. The methanol extract was filtered, concentrated *in vacuo* at approximately 40°C, and dissolved in 1 mL of 50% methanol. The resultant solution (10 μL) was analyzed by high-performance liquid chromatography (HPLC; Prominence UFLC series, Shimadzu, Kyoto, Japan) with analysis of spectral characteristics using a diode array detector (DAD; SPDM20A, Shimadzu) according to the method described previously [35]. For the quantification of phenolic compounds in root extracts, the spectral characteristics from 220 to 400 nm and the retention times of chlorogenic acid (MP Biomedicals LLC., Santa Ana, CA, USA) were compared with those of the peaks in the root extracts. To measure the molecular weight of phenolic compounds in the root extracts, a high performance liquid chromatography/electrospray ionization-mass spectrometer (HPLC/ESI-MS; LC/MS-2020 series, Shimadzu) equipped with a UV-VIS detector (SPD-20A; Shimadzu) at 320 nm was used. Nitrogen was used as the nebulizer gas (N_2_ supplier 24F; System instruments, Tokyo, Japan), and MS was operated in the total ion count mode (scanning range, *m/z* 50–500). The HPLC conditions were as follows: column, Mightysil RP-18 MS (150 × 2.0 mm; Kanto, Tokyo, Japan); eluent, aq. 0.1% formic acid (solvent A) and 100% acetonitrile (solvent B); flow rate, 0.2 mL/min at 40°C. The following gradient was used for the eluent system: 0–10 min, 70% A and 30% B; 10–20 min, 50% A and 50% B; and 20–40 min, 100% B. For the quantification of phenolic compounds, an absolute calibration curve of chlorogenic acid was prepared by HPLC-DAD at 320 nm. The results of five replications were averaged, and SEs were calculated.

Roots of *P*. *densiflora* seedlings used for elemental analysis described above were also employed for the analysis of productions in the roots of the seedlings. Among the eight seedlings, four seedling roots were used for the analysis of phenolic compounds, including condensed tannins, and the others were used for organic acids analysis. Phenolic compounds analysis was performed using the methods described above. For the quantification of phenolic compounds in root extracts, the spectral characteristics from 220 to 400 nm and the retention times of catechin (MP Biomedicals LLC., Santa Ana, CA, USA) were compared with those of the peaks in the root extracts. The molecular weights of the phenolic compounds in the root extracts were measured via HPLC/ESI-MS equipped with a UV-VIS detector at 280 nm. HPLC/ESI-MS analysis was conducted as previously described. For the quantification of phenolic compounds, an absolute calibration curve of catechin was prepared by HPLC-DAD at 280 nm. The results of four replications were averaged, and SEs were calculated.

Condensed tannins were quantified according to the method described in [36]. The butanol reagent was prepared as follows: 0.7 g FeSO_4_·7H_2_O and 50 mL of HCl (36%) were mixed and filled up to 1000 mL with butanol. The samples (300 μL) used for HPLC-DAD analysis were added to 3.5 mL of butanol reagent, and reacted at 90°C for 40 min. The absorbance of the reactants was measured at 550 nm using a UV-VIS detector (UV-2450, Shimadzu). Condensed tannin concentrations were calculated using a cyanidin chloride standard curve (Wako Pure Chemical Industries Ltd., Osaka, Japan). The concentration of the condensed tannins was expressed as cyanidin chloride equivalents. The results of four replications were averaged, and SEs were calculated.

For organic acid analysis, the roots of four *P*. *densiflora* seedlings were extracted in 80% ethanol for 5 days at 23°C in the dark. The extract was filtered, concentrated *in vacuo* at approximately 40°C and dissolved in 200 μL of 50% methanol. The resultant solution was applied to an anion exchange column (TOYOPAK DEAE M, Tosoh Corporation, Shunan, Japan), and the organic acids were eluted with 6 mol/L formic acid. The eluate was freeze-dried (VD-250F; Taitec, Saitama, Japan) to remove formic acid, and the residue was dissolved in 100 μL of pyridine. Then, 100 μL of *N*-methyl-*N*-(trimethylsilyl) trifluoroacetamide (MSTFA; Thermo Scientific, Bellefonte, PA, USA) was added, and the sample was trimethylsilylated at 37°C for 30 min. The organic acid concentration was measured using gas chromatography-mass spectrometry (GC-MS) on a QP2010 instrument equipped with a GC-2010 electron-ionization mass spectrometry detector (Shimadzu) and a low-polar InertCap 5MS/Sil capillary column (30 m × 0.25 mm i.d., 0.25-μm film thickness; GL Sciences Inc., Tokyo, Japan) following the methods described in [37]. The mass spectral characteristics at *m/z* 50–500 and retention times of malic acid (Wako Pure Chemical Industries Ltd.)-trimethylsilyl were compared with those of the peaks in the root extracts. For the quantification of organic acids, the absolute calibration curve of malic acid-trimethylsilyl was measured using the selected ion mode (*m/z* 73, 147, and 233). The results of four replications were averaged, and SEs were calculated.

### Infection rate and isolation of root endophytes from *M*. *sinensis* and *P*. *densiflora* seedlings

In September, five *M*. *sinensis* individuals and four current-year seedlings of *P*. *densiflora* outside and inside the patches were collected. A part of the root was used to calculate infection rates, and the other part of the root was used for the isolation of root endophytes. The collected roots were washed with deionized water and stained with trypan blue as previously described [38]. The trypan-blue-stained roots were observed by microscopy (CX21, Olympus) to calculate the infection rate of microbes as follows: arbuscular mycorrhiza (AM mycorrhiza; *Paris*-type; [39]), root endophytes (microsclerotia; [40]), and ectomycorrhiza (hartig net; [41]). Infection rates were calculated according to [42,43]. The results were averaged, and SEs were calculated (*M*. *sinensis*, n = 5; *P*. *densiflora*, n = 4).

For the isolation of root endophytes, roots of *M*. *sinensis* were surface-sterilized with 70% ethanol for 1 min, 7.5% hydrogen peroxide solution for 5 min, and again with 70% ethanol for 1 min. For *P*. *densiflora* seedlings outside and inside the patches, the roots were surface-sterilized with 70% ethanol for 1 min, 15% hydrogen peroxide solution for 5 min, and again with 70% ethanol for 1 min. The roots were rinsed twice with sterile deionized water for 5 min to remove the reagents and were dried on sterile filter paper on a clean bench for 5 min. Sterilized roots were cut into approximately 10-mm segments with a sterile scalpel, and 100 segments were randomly cut from each plant sample. Totally, 500 segments of *M*. *sinensis* and 400 segments of *P*. *densiflora* seedlings outside and inside the patches were placed on 1% malt extract and incubated at 23°C in the dark for 2 weeks. Isolated fungal species were microscopically observed and purified. The root endophyte detection rate (%) for each fungus was calculated using the following formula:

$$Root\;endophyte\;detection\;rate=\frac{N_{d}}{N_{t}}\times 100$$ (3)

Where N_d_ is the number of root segments from which the fungus was detected, and N_t_ is the total number of root segments used for fungal isolation.

The genera of the most frequent isolates were identified by morphological observation. Root endophytes detected at high frequencies were identified using morphological characteristics and molecular analysis. DNA templates were prepared from a small piece of mycelial mass, crushed in 50 μL of sterilized water, and heated for 15 s in a microwave oven. The ITS regions were amplified using the primers ITS5 and ITS4 [44]. The PCR conditions included an initial denaturing step at 94°C for 4 min, 35 cycles at 94°C for 30 s, 52°C for 50 s, and 72°C for 50 s, and a final elongation at 74°C for 6 min. The reaction mixture included 25 μL of GoTaq master mix (Promega Co., Ltd., Madison, WI, USA), 10 pmol of each primer, and 1 μL of DNA template. Amplicons were purified with the QIAquick PCR Purification Kit (Qiagen, Hilden, Germany), sequenced with a BigDye Terminator Cycle Sequencing FS Ready Reaction Kit ver. 3.1, and analyzed using an ABI3100 genetic analyzer (Applied Biosystems, Carlsbad, CA, USA). For molecular identification, the sequences were subjected to BLAST comparisons in the National Center for Biotechnology Information database (http://www.ncbi.nlm.nih.gov/).

### Statistical analysis

Statistical analyses were performed using IBM-SPSS Statistics software for Windows (ver. 26.0.0.1, IBM, Armonk, NY, USA). Differences in temperature, diurnal range, water content, pH (H_2_O), harmful metal concentrations, and available Fe concentration in the soil outside and inside the patches were evaluated using the Student’s t-test. Differences in infection rates of root endophytes and concentrations of harmful metals, nutrient elements, and harmful metal detoxicants in *P*. *densiflora* seedlings were evaluated using Student’s t-test. Differences were considered statistically significant at P < 0.05. The water content in the soil and infection rates of the root endophytes were statistically analyzed after arcsine transformation.

The concentrations of harmful metals in *M*. *sinensis* and *P*. *densiflora* seedling tissues and transfer factors of harmful metals in *M*. *sinensis* were evaluated using one-factor analysis of variance (one-factor ANOVA, Scheffé post hoc test, P < 0.05).

## Results

### Survival rates and mortality factors of *P*. *densiflora* seedlings

**Fig 2:**
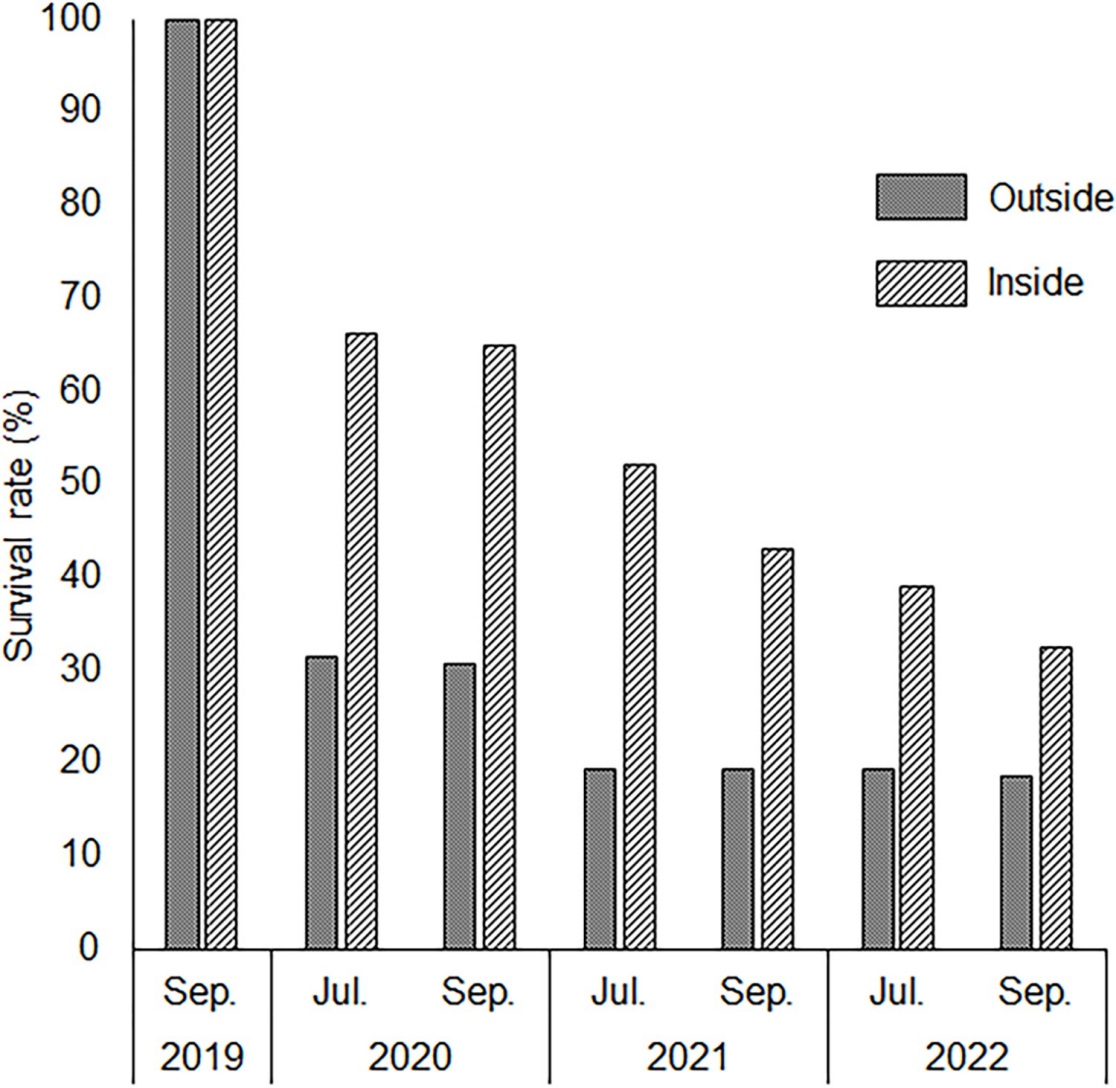
Survival rates of *Pinus densiflora* seedlings outside and inside the patches September 2019–September 2022. Seedlings of *P*. *densiflora* outside and inside the patches were identified in September 2019.

From September 2019 to July 2020, the survival rates of *P*. *densiflora* seedlings outside and inside the patches decreased sharply to approximately 30% and 65%, respectively (Fig 2). Throughout the sampling period, the survival rates of *P*. *densiflora* seedlings inside the patches were higher than those of seedlings outside the patches (Fig 2). There were no significant differences in mortality factors of the *P*. *densiflora* seedlings outside and inside the patches; root diseases were the primary mortality factors (S1 Fig). *Ceratobasidium bicorne* was isolated from the roots of dead seedlings of *P*. *densiflora* outside patches as a possible pathogen (S1 Table). Dark-septate endophytes (DSEs) were frequently isolated from the roots of dead seedlings of *P*. *densiflora* outside and inside the patches (S1 Table).

### Harmful metal concentrations, pH (H_2_O), water content, and temperature in soil outside and inside the patches

**Fig 3:**
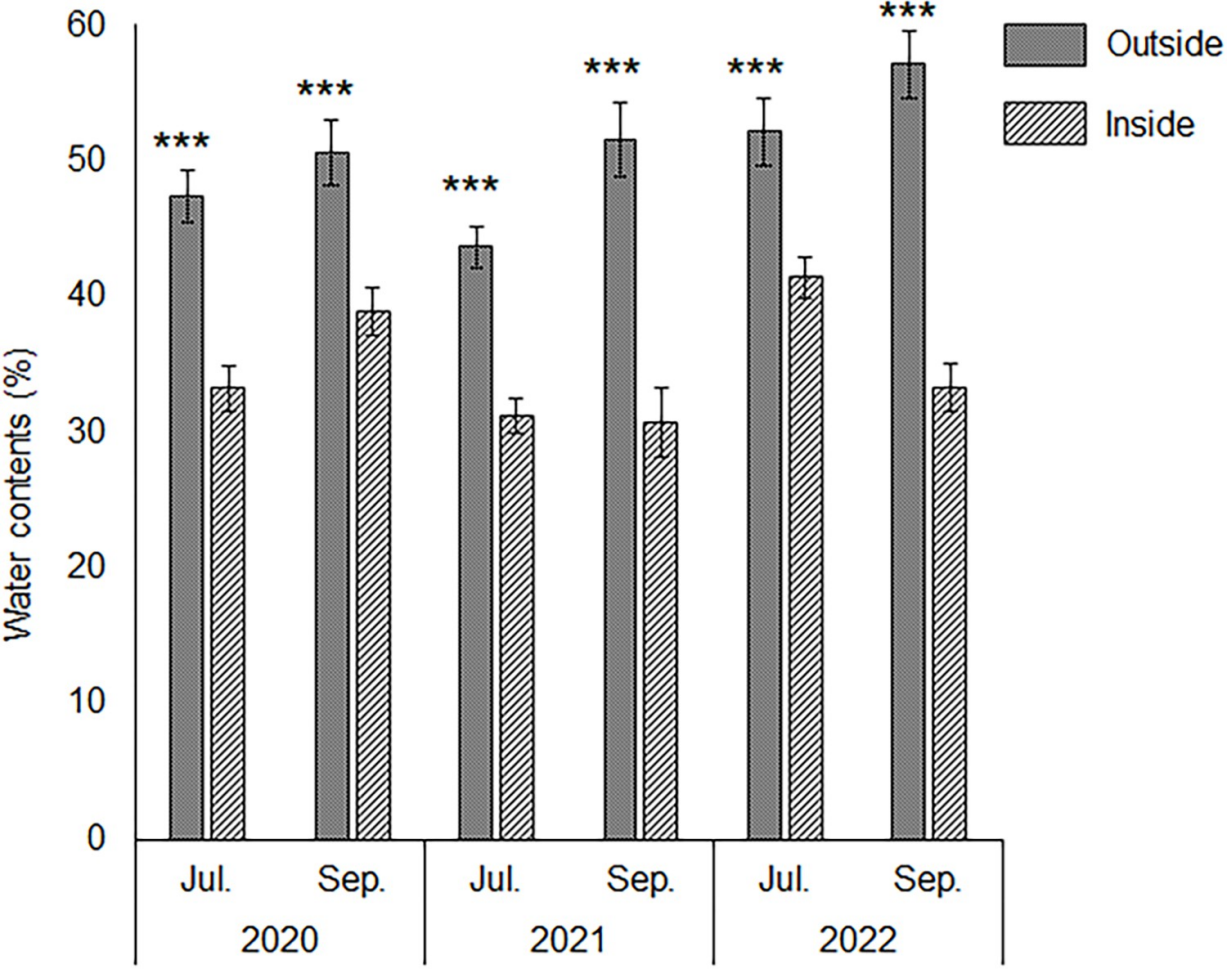
Water content in soils outside and inside the patches. Differences between treatments were evaluated using the Student’s t-test (*** P < 0.001, n = 20). Error bars represent standard error (SE).

Soils outside and inside the patches contained high concentrations of Fe (Table 1) compared with those in common soils (40 g/kg dry weight (DW) [17]). There were no significant differences in harmful metal concentrations or pH (H_2_O) between soils outside and inside the patches (P > 0.05; Table 1). Throughout the sampling period, the water content of the soils inside the patches was significantly lower than that outside the patches (P < 0.001; Fig 3). The average of soil temperature outside the patches was significantly higher than that inside the patches (P < 0.001; Table 2). The highest/lowest soil temperatures were 33.8°C/19.4°C and 29.1°C/21.4°C outside and inside the patches, respectively (Table 2; S2 Fig) The average of diurnal range of soil temperature outside the patches was significantly larger than that inside the patches (P < 0.001; Table 2, S2 Fig).

**Table 1:** Harmful metal concentrations and pH (H_2_O).

Element (g/kg DW)	Outside soil	Inside soil
Al	52.9 ± 5.9	60.4 ± 2.3
Cu	10.7 ± 1.2	11.4 ± 0.5
Fe	165.5 ± 18.3	198.5 ± 8.9
Mn	1.5 ± 0.2	1.9 ± 0.1
Zn	19.0 ± 2.0	19.2 ± 1.1
Available Fe	N. D.	N. D.
pH (H_2_O)	7.73 ± 0.02	7.51 ± 0.09

DW; dry weight. There were no significant differences between the outside and inside patches using the Student’s t-test (P > 0.05, n = 4). Results are expressed as mean ± standard error (SE).

**Table 2:** Soil temperature outside and inside the patches.

	Outside (°C)	Inside (°C)
Average	25.2 ± 0.05***	24.4 ± 0.03
Maximum	33.8	29.1
Minimum	19.4	21.4
Diurnal range	5.2 ± 0.2***	2.1 ± 0.1

Differences between treatments were evaluated using the Student’s t-test (*** P < 0.001, average of soil temperature, n = 2642; average of diurnal range, n = 112). Results are expressed as mean ± standard error (SE).

### Harmful metal and nutrient element concentrations in *M*. *sinensis* and *P*. *densiflora* seedlings

**Fig 4:**
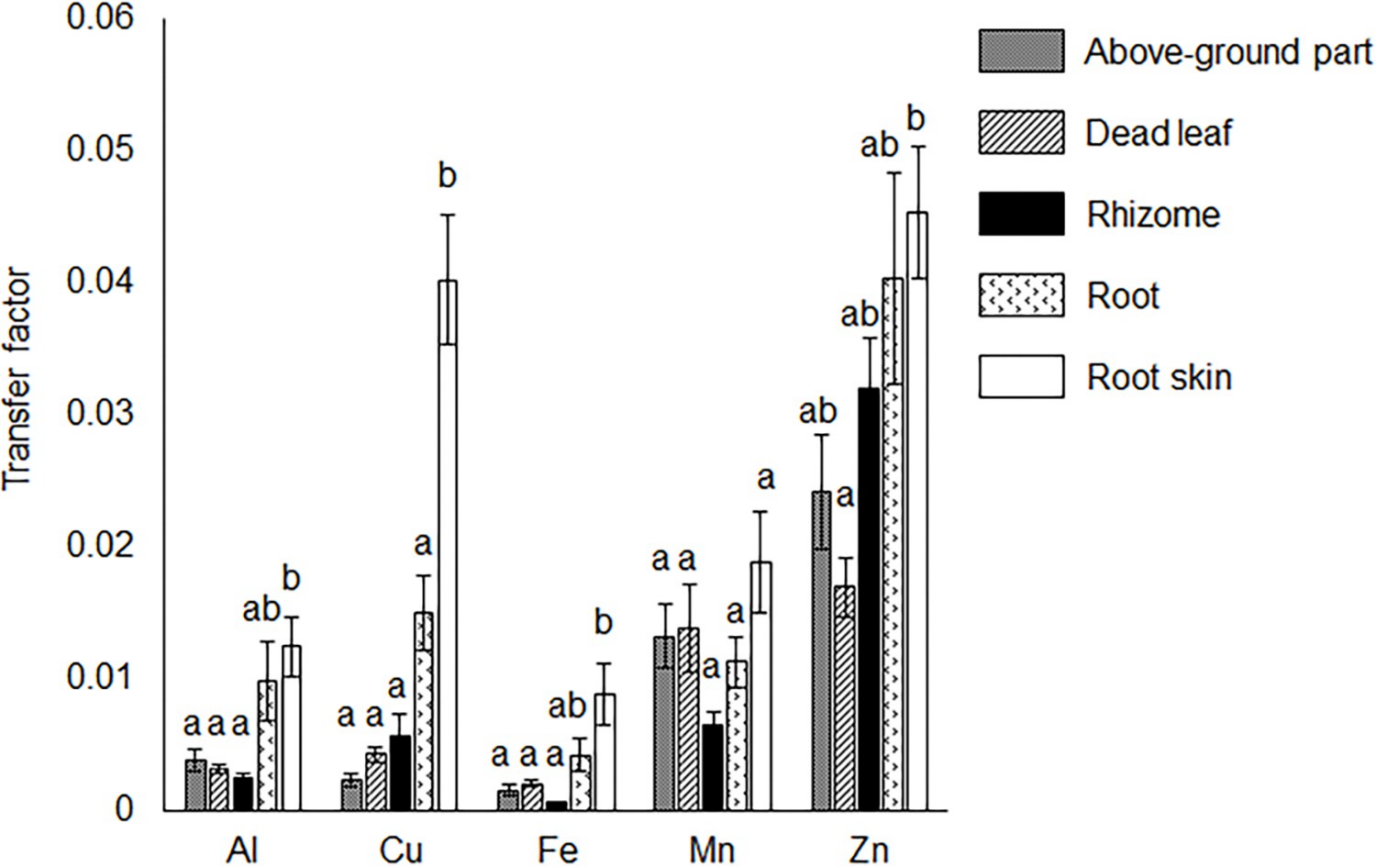
Transfer factors in *Miscanthus sinensis* tissues. The transfer factors of harmful metals in *M*. *sinensis* (ratio of concentration in each tissue of *M*. *sinensis* to the soil concentration) were calculated. Different letters indicate statistically significant differences among treatments in one-factor ANOVA, Scheffé post-hoc test, P < 0.05 (n = 5). Error bars represent standard error (SE).

Although available Fe was not detected in the soils, *M*. *sinensis* contained markedly higher concentrations of Fe in the roots and root skins than other harmful metals (P < 0.05; Table 3A). *Miscanthus sinensis* contained high concentrations of Ca in all tissues (Table 3B), and the transfer factors of Al, Cu, Fe, and Zn were significantly higher in the root skin than in the other tissues (P < 0.05; Fig 4).

**Table 3:** Harmful metal and nutrient element concentrations in *Miscanthus sinensis*.

Element (mg/kg DW)	Above-ground part	Dead leaf	Rhizome	Root	Root skin
(a)					
Al	216.1 ± 41.8ab	183.6 ± 14.6bc	147.1 ± 16.6a	568.1 ± 172.3ab	723.1 ± 127.3ab
Cu	25.2± 4.4a	46.0 ± 4.5ab	61.5 ± 18.4a	168.2 ± 36.5ab	435.7 ± 44.6a
Fe	288.0± 64.8bc	395.7 ± 44.4d	121.3 ± 14.4a	842.0 ± 280.5b	1661.2 ± 424.1b
Mn	24.7 ± 4.6a	25.1 ± 5.2a	12.2 ± 1.9a	21.3 ± 4.2a	34.6 ± 7.0a
Zn	448.5 ± 69.5c	320.6 ± 44.1cd	596.7 ± 57.0b	746.6 ± 130.3ab	848.7 ± 82.8ab
(b)					
Ca	18076.5 ± 2633.1a	14758.2 ± 1719.3a	15840.9 ± 1058.9a	21430.8 ± 4392.2a	18267.0 ± 1191.8a
K	9211.0 ± 739.2b	1553.8 ± 251.9b	5374.9 ± 1089.4b	1958.2 ± 324.6b	311.5 ± 10.8b
Mg	2209.5 ± 182.9c	1416.2 ± 166.8b	1629.9 ± 126.6c	905.5 ± 110b	961.0 ± 161.5b
P	708.2 ± 68.3c	206.5 ± 8.9b	392.5 ± 75.9c	188.0 ± 11.3b	88.6 ± 6.5b

DW; dry weight. Different letters indicate statistically significant differences among treatments in one-factor ANOVA, Scheffé post-hoc test, P < 0.05 (n = 5). Results are expressed as mean ± standard error (SE).

*Pinus densiflora* seedlings outside and inside the patches contained high concentrations of Al and Fe in their roots (Table 4). Concentrations of Fe were significantly higher than those of other harmful metals (P < 0.05; S2 Table). In September 2020, hypocotyls inside the patches contained significantly higher concentrations of Zn than those inside the patches (P < 0.01; Table 4). In September 2021, hypocotyls of *P*. *densiflora* seedlings inside the patches contained lower Mn concentrations than those of seedlings outside patches (P < 0.01; Table 4). In terms of nutrient element concentrations, in July 2020, *P*. *densiflora* seedlings inside the patches contained significantly higher concentrations of K in leaves, hypocotyls, and roots than those in seedlings outside the patches, and *P*. *densiflora* seedling roots inside the patches contained significantly lower concentrations of Mg than those of seedlings outside the patches (P < 0.05; Table 5). In September 2020, *P*. *densiflora* seedlings inside the patches contained significantly lower Ca and Mg concentrations in leaves (P < 0.05) and Mg concentrations in hypocotyls (P < 0.01) than those of seedlings outside the patches (Table 5). In July 2021, *P*. *densiflora* seedlings inside the patches contained significantly lower Ca concentrations in leaves and hypocotyls (P < 0.05) and significantly higher K concentrations in hypocotyls (P < 0.001) than seedlings outside the patches (Table 5). In September 2021, *P*. *densiflora* seedlings inside the patches contained significantly lower Ca concentrations in the leaf and hypocotyl (P < 0.05) and significantly higher hypocotyl K concentrations (P < 0.001) than the seedlings outside the patches (Table 5).

**Table 4:** Harmful metal concentrations in *Pinus densiflora* seedlings outside and inside the patches.

Element (mg/kg DW)	Leaf		Hypocotyl		Root	
	Outside	Inside	Outside	Inside	Outside	Inside
Jul. 2020						
Al	83.2 ± 8.4	120.3 ± 16.9	184.2 ± 24.9	183.1 ± 29.7	4486.7 ± 275.5	4556.7 ± 743.5
Cu	12.2 ± 2.6	13.3 ± 2.6	33.0 ± 5.4	48.2 ± 11.7	1121.7 ± 82.9	1389.0 ± 218.6
Fe	122.1 ± 30.9	144.7 ± 42.3	274.1 ± 64.8	324.8 ± 70.5	8002.7 ± 580.5	9157.4 ± 1764.4
Mn	51.2 ± 6.5	43.5 ± 6.7	20.3 ± 1.6	15.4 ± 2.0	99.6 ± 9.4	86.5 ± 10.8
Zn	123.4 ± 13.8	169.1 ± 38.2	168.0 ± 18.9	202.0 ± 30.3	1302.9 ± 89.3	1712.4 ± 197.2
Sep. 2020						
Al	116.8 ± 15.0	173.1 ± 51.2	187.5 ± 12.4	204.1 ± 20.2	2893.4 ± 210.6	2634.7 ± 228.2
Cu	19.9 ± 1.9	29.2 ± 9.6	167.1 ± 118.5	85.3 ± 9.9	868.4 ± 87.3	959.2 ± 83.1
Fe	208.5 ± 25.3	394.3 ± 160.5	286.8 ± 24.9	387.9 ± 62.3	5065.4 ± 505.1	5752.5 ± 502.8
Mn	51.8 ± 6.2	39.5 ± 4.5	17.8 ± 1.4	15.5 ± 0.7	100.6 ± 16.2	70.9 ± 8.8
Zn	147.3 ± 17.1	215.3 ± 32.9	177.3 ± 15.1	330.6 ± 38.7**	900.2 ± 78.5	1175.7 ± 154.5
Jul. 2021						
Al	92.8 ± 30.3	61.5 ± 20.2	398.5 ± 43.7	278.5 ± 74.1	6085.9 ± 531.7	4798.0 ± 930.1
Cu	28.3 ± 11.2	12.2 ± 8.1	231.6 ± 62.0	207.5 ± 82.0	2718.7 ± 442.7	2228.9 ± 382.3
Fe	195.6 ± 83.4	118.3 ± 35.1	625.7 ± 86.0	502.8 ± 118.9	7842.5 ± 963.8	8143.8 ± 1667.5
Mn	19.5 ± 4.0	16.2 ± 2.5	28.7 ± 3.4	24.3 ± 4.6	145.3 ± 12.3	157.5 ± 38.8
Zn	101.9 ± 45.8	89.8 ± 11.8	407.9 ± 75.8	344.2 ± 62.1	1914.9 ± 244.6	2009.8 ± 317.4
Sep. 2021						
Al	132.1 ± 31.2	65.7 ± 19.6	434.4 ± 114.7	198.4 ± 21.4	4565.2 ± 601.5	3826.1 ± 395.9
Cu	17.8 ± 6.5	7.9 ± 1.9	177.7 ± 47.5	101.9 ± 22.0	1645.5 ± 195.8	2014.4 ± 184.9
Fe	247.9 ± 67.2	147.2 ± 38.1	878.8 ± 287.2	293.0 ± 35.7	7302.8 ± 1061.3	6504.7 ± 1108.4
Mn	32.7 ± 5.4	22.5 ± 6.1	22.8 ± 4.3**	8.5 ± 1.3	121.0 ± 16.3	116.0 ± 19.3
Zn	128.5 ± 15.7	139.0 ± 29.0	371.8 ± 75.0	257.4 ± 37.3	1435.9 ± 180.7	1770.0 ± 161.7

DW; dry weight. Differences between treatments were evaluated using the Student’s t-test (** P < 0.01, n = 8). Results are expressed as mean ± standard error (SE).

**Table 5:** Nutrient element concentrations in *Pinus densiflora* seedlings outside and inside the patches.

Element (mg/kg DW)	Leaf		Hypocotyl		Root	
	Outside	Inside	Outside	Inside	Outside	Inside
Jul. 2020						
Ca	7793.2 ± 674.6	7438.4 ± 960.0	3830.4 ± 400.0	3856.2 ± 490.3	11381.5 ± 793.0	11045.7 ± 845.4
K	3563.7 ± 465.5	5425.4 ± 485.6*	2637.7 ± 241.3	3635.0 ± 369.4*	3073.6 ± 375.2	4967.3 ± 726.4*
Mg	1166.5 ± 76.2	1281.5 ± 120.4	1033.9 ± 137.6	861.2 ± 113.7	1734.7 ± 108.5*	1470.3 ± 49.5
P	1084.4 ± 99.1	1072.7 ± 222.3	1433.2 ± 186.5	1359.5 ± 410.1	1366.9 ± 91.0	1589.8 ± 330.8
Sep. 2020						
Ca	10920.5 ± 923.1*	7888.9 ± 961.0	3937.9 ± 413	3829.4 ± 351.9	8994.1 ± 1109.7	9575.5 ± 2054.6
K	4985.2 ± 599.2	6088.8 ± 675.1	3702.0 ± 182.6	4405.5 ± 380.3	2508.1 ± 316.5	2767.7 ± 192.8
Mg	1671.2 ± 151.9*	1255.6 ± 73.9	778.5 ± 38.9**	614.8 ± 20.2	1154.2 ± 87.7	956.6 ± 56.6
P	965.3 ± 198.4	699.1 ± 41.8	992.1 ± 165.5	790.2 ± 41.9	913.9 ± 120.9	761.4 ± 38.8
Jul. 2021						
Ca	5163.8 ± 691.4*	2975.3 ± 286.5	8219.9 ± 506.8**	5314.6 ± 717.4	26717.5 ± 3974.2	18259.4 ± 2883.9
K	6373.8 ± 554.9	5910.7 ± 1019.0	4580.6 ± 443.6	7087.5 ± 1287.1	2951.1 ± 519.1	3411.7 ± 327.7
Mg	871.3 ± 107.5	785.5 ± 69.0	736.1 ± 54.6	713.5 ± 44.3	1505.2 ± 103.6*	1187.7 ± 95.0
P	416.7 ± 43.7	373.0 ± 16.0	456.6 ± 37.6	412.3 ± 31.9	606.0 ± 28.1	607.2 ± 48.1
Sep. 2021						
Ca	9954.7 ± 1265.1*	5634.2 ± 738.2	6998.4 ± 553.6*	5184.1 ± 466.3	22211.5 ± 2883.7	21715.5 ± 2753.1
K	3788.1 ± 471.9	4756.8 ± 485.0	2844.3 ± 273.7	4491.3 ± 165.7***	1099.4 ± 158.6	1963.6 ± 418.3
Mg	1756.8 ± 178.7	1324.5 ± 128.0	887.8 ± 90.9	763.4 ± 47.9	1517.6 ± 174.0	1517.1 ± 79.3
P	352.7 ± 31.4	385.1 ± 32.8	379.7 ± 31.3	463.6 ± 48.4	409.8 ± 66.1	389.1 ± 18.7

DW; dry weight. Differences between treatments were evaluated using the Student’s t-test (* P < 0.05, ** P < 0.01, *** P < 0.001, n = 8). Results are expressed as mean ± standard error (SE).

### Fe detoxicants in roots of *M*. *sinensis* and *P*. *densiflora* seedlings

HPLC/ESI-MS analysis of the phenolic compounds in *M*. *sinensis* roots detected *m/z*: 355 ([M+H]^+^), *m/z*: 377 ([M+Na]^+^), *m/z*: 393 ([M+K]^+^), and *m/z*: 353 ([M-H]^-^), resulting in a molecular weight of 354. HPLC/ESI-MS and HPLC-DAD analyses revealed that the phenolic compound was chlorogenic acid. The concentration of chlorogenic acid was 1.23 ± 0.20 μg/mg fresh weight (FW) in *M*. *sinensis* roots.

**Fig 5:**
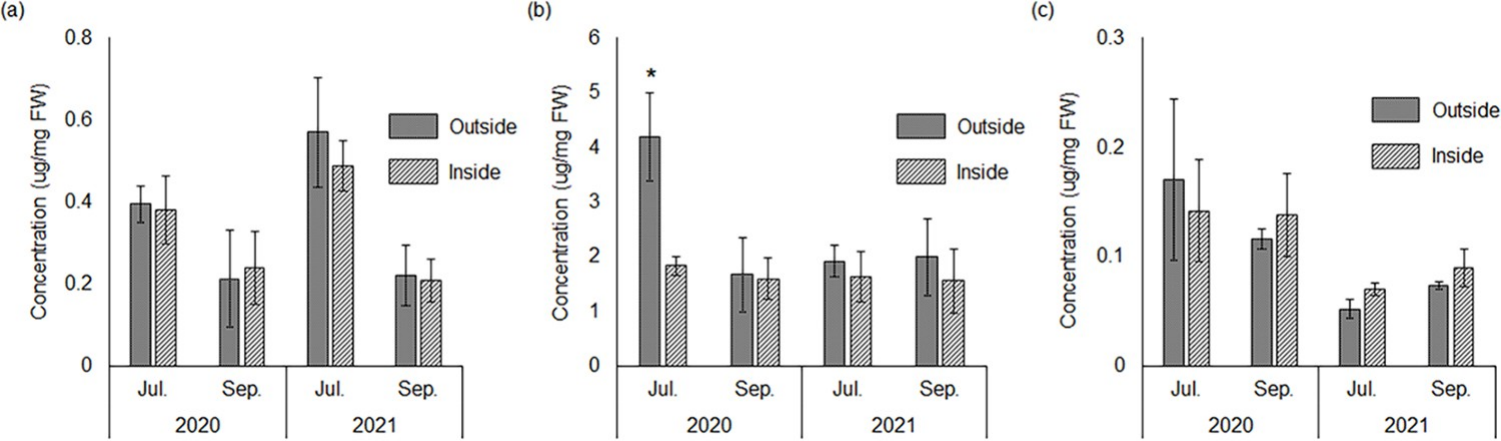
Concentrations of phenolic compounds and organic acids in roots of *Pinus densiflora* seedlings. (a) Concentration of catechin, (b) concentration of condensed tannin, and (c) concentration of malic acid. The concentrations of condensed tannin were expressed as cyanidin chloride equivalents. Differences between treatments were evaluated using Student’s t-test (* P < 0.05, n = 4). FW: fresh weight; error bars represent ± standard error (SE).

HPLC/ESI-MS analysis of the phenolic compounds in the roots of *P*. *densiflora* seedlings detected *m/z* of 291 ([M+H]^+^) and *m/z* of 289 ([M-H]^-^), resulting in a molecular weight of 290. HPLC/ESI-MS and HPLC-DAD analyses revealed that the phenolic compound was catechin. In July 2020, *P densiflora* seedlings outside the patches contained significantly higher concentrations of condensed tannins than those inside the patches (P < 0.05; Fig 5B). GC/MS analysis showed that *P*. *densiflora* seedlings produced malic acid. The concentrations of each compound in the roots of *P*. *densiflora* seedlings are shown in Fig 5.

### Infection rate and detection rates of root endophytes in *M*. *sinensis* and *P*. *densiflora*

**Fig 6:**
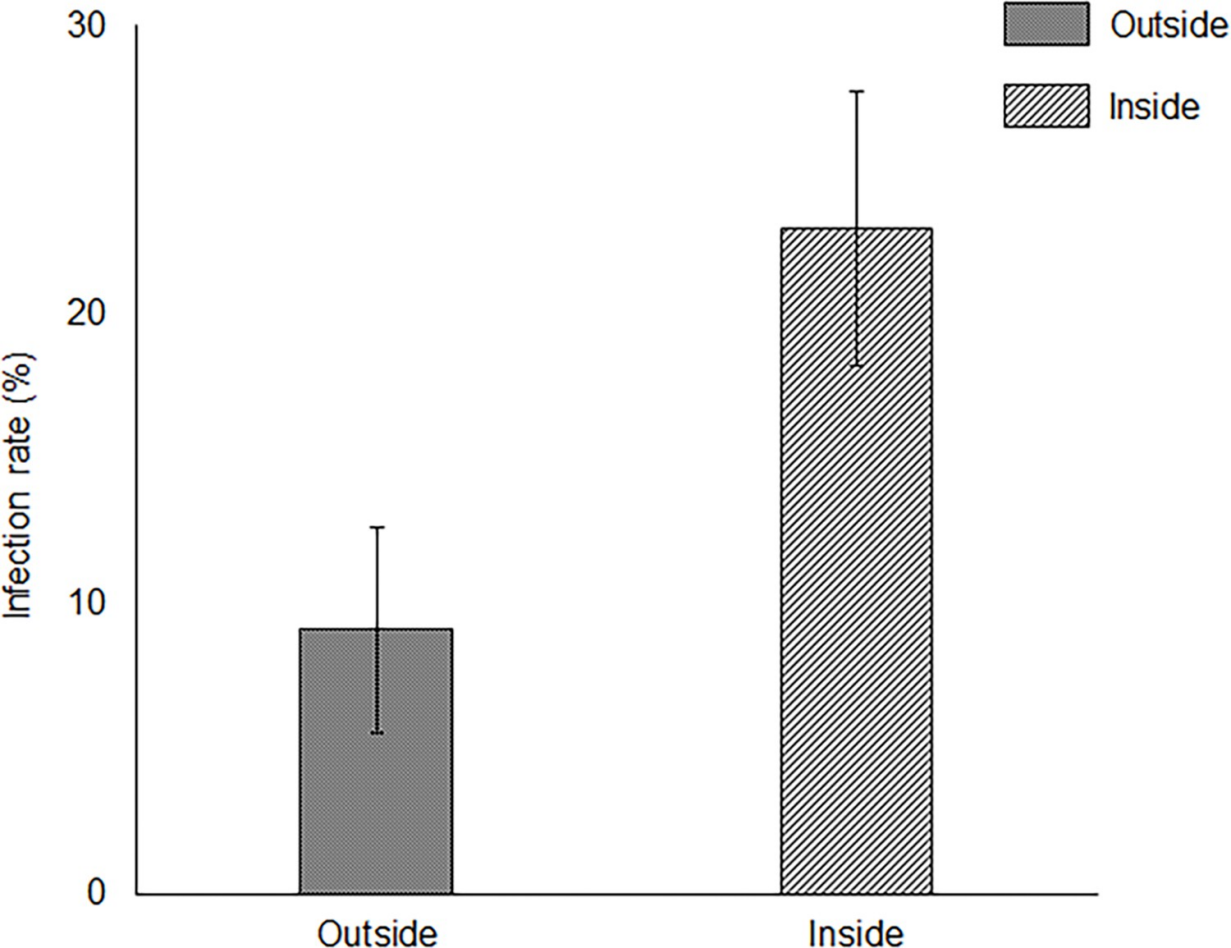
Infection rate of root endophytes outside and inside the patches. Results are expressed as mean ± standard error (SE). There were no significant differences between the outside and inside patches using the Student’s t-test (P > 0.05, n = 4). Error bars represent standard error (SE).

Microscopic observation of trypan-blue-stained roots of *M*. *sinensis* revealed that the infection rates of AM mycorrhiza and endophyte were 1.9 ± 0.5% and 39.3 ± 3.6%, respectively. Root endophyte infection was observed in *P*. *densiflora* seedlings both outside and inside the patches (Fig 6). In contrast, infection by AM mycorrhiza and ectomycorrhiza was not observed (Fig 6).

**Fig 7:**
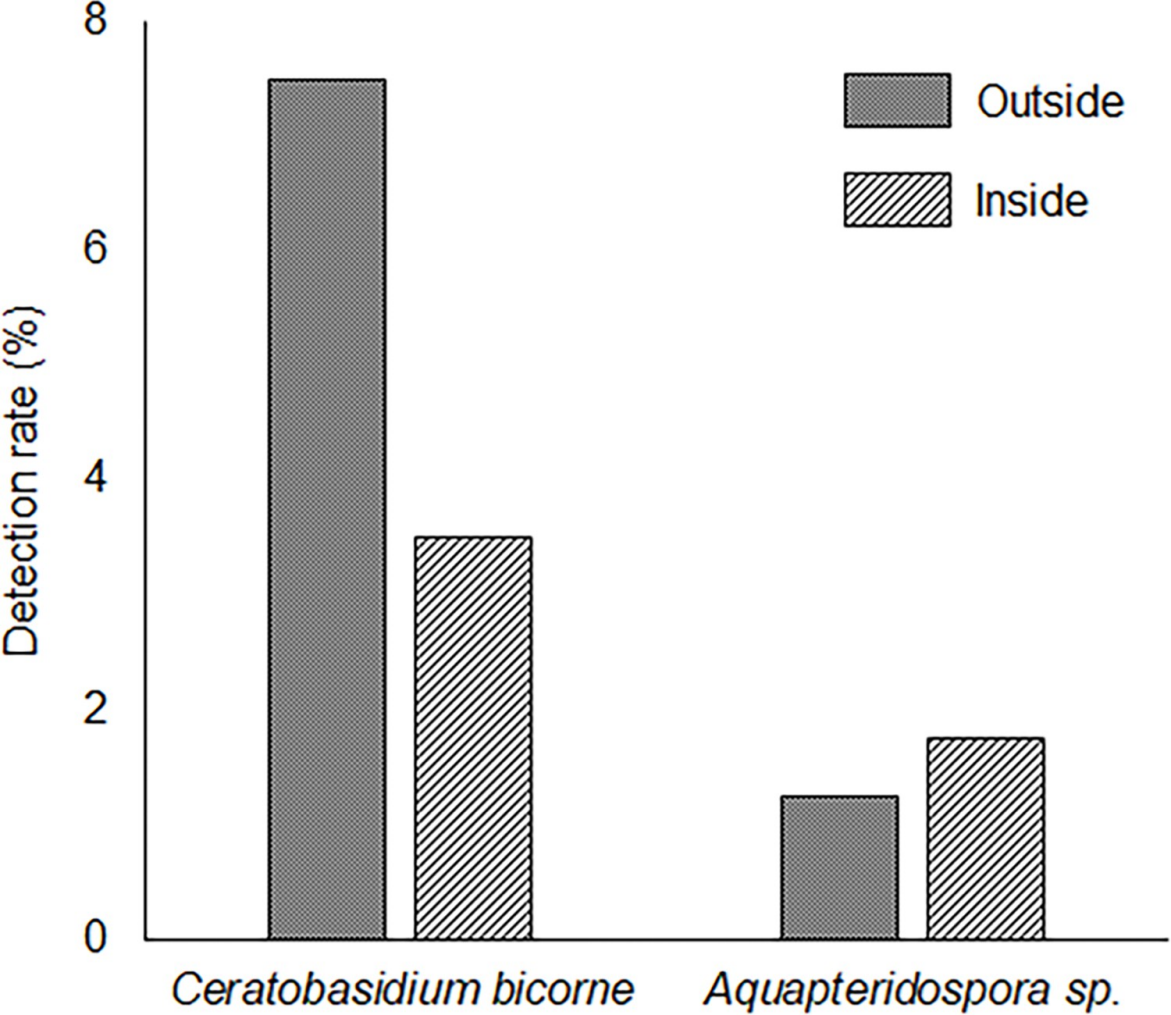
Detection rate of root endophytes isolated from *Pinus densiflora* seedlings outside and inside the patches. Four *P*. *densiflora* seedlings outside and inside the patches were used to isolate root endophytes. Root endophytes were isolated from 100 sections per each seedling.

The three genera of root endophytes frequently isolated from roots of *M*. *sinensis* were *Magnaporthaceae* sp. (detection rate, 9.8%), *Cladophialophora* sp. (9.0%), and *Aquapteridospora* sp. (7.2%). The two genera of root endophytes isolated at a frequency higher than 1% from the roots of *P*. *densiflora* seedlings outside and inside the patches were *C*. *bicorne* and *Aquapteridospora* sp. (Fig 7). Several root endophytes with melanized dark hyphae and septate are classified as DSEs [45,46]. *Aquapteridospora* sp. had dark hyphae and septate, indicating that the root endophyte is considered as a DSE.

## Discussion

At the study site, plants were exposed to high soil temperatures and heavy metal stress. *Miscanthus sinensis* formed patches to improve *P*. *densiflora* survival in our study site (Fig 2). Environmental factors influenced by pioneer species, such as *M*. *sinensis* include mitigation of strong wind disturbance [17], alleviation of heavy metal toxicity [47,48], supply of nutrient elements such as P [49], soil moisture retention [50,51], suppression of soil temperature alteration [52], and pH buffering capacity [53]. These functions of *M*. *sinensis* would improve environmental conditions, resulting in increased *P*. *densiflora* survival rates. *Miscanthus sinensis* contained high concentrations of Fe in its roots, and Fe tolerance mechanisms such as the production of chlorogenic acid and exclusion mechanisms such as root skin turnover were observed, which was consistent with our previous report [20]. Pioneer species with high turnover rates provide litter and improve the soil environment [48], implying that *M*. *sinensis* can improve the soil conditions inside the patches. Harmful metals were not transferred into the aboveground parts of *M*. *sinensis* (Fig 4), which suggested that *M*. *sinensis* would provide litter containing low concentrations of harmful metals to the soil. However, there were no significant differences between harmful metal concentrations outside and inside the patches (Table 1), indicating that *M*. *sinensis* could not decrease the concentrations of harmful metals in the soil. There were no differences in pH (H_2_O) between the outside and inside patches (Table 1). These results indicate that the soil properties mentioned above did not affect the survival rate of *P*. *densiflora* seedlings. Soil water content (Fig 3), average of soil temperature, and diurnal range (Table 2, S2 Fig) showed significant differences between the outside and inside the patches. *Pinus densiflora* can ordinally grow at 30% water content in soil [54], and an average of soil temperature within 22°C±5°C cannot inhibit the plant growth [55–57]. Therefore, water content and average soil temperature would not affect *P*. *densiflora* seedling survival at the study site. In contrast, a large diurnal range in soil temperature damages root systems and inhibits the growth of *Capsicum annum* [52]. In addition, sharp changes in soil temperature inhibit root growth [58], and root respiration decreases from 32°C to 35°C [55]. Because vegetation was not observed outside the patches (Fig 1), sunlight could directly reach the soil and raise the soil temperature to 33.8°C (Table 2; S2 Fig). These results indicated that the outside of the patches would not be suitable for *P*. *densiflora* seedling survival. Therefore, *M*. *sinensis* would suppress the excessive increase and sharp alternation of soil temperature to reduce environmental stress in *P*. *densiflora* seedlings, which facilitated the establishment of *P*. *densiflora* and enhanced vegetation succession.

Plants naturally growing in heavy metal environments can adapt to heavy-metal stress [15–17]. Seedlings of *P*. *densiflora* outside and inside the patches contained particularly high concentrations of Fe among the harmful metals (P < 0.05; S2 Table), indicating that *P*. *densiflora* seedlings would show Fe tolerance. There were no differences between the concentrations of harmful metals in *P*. *densiflora* seedlings outside and inside the patches (Table 4). These results suggested that *M*. *sinensis* would not suppress *P*. *densiflora* seedlings to absorb Fe via competition of Fe uptake between *M*. *sinensis* and *P*. *densiflora* seedlings. *Pinus densiflora* seedlings outside and inside the patches produce catechin, condensed tannin, and malic acid, which chelate and detoxify Fe [59–61] to acquire Fe tolerance. Condensed tannin production is enhanced by high temperatures [62]. In July 2020, condensed tannin production was enhanced in *P*. *densiflora* seedlings outside the patches (Fig 5), suggesting that the seedlings outside the patches were exposed to high soil temperature stress, as soil temperatures outside the patches were more easily elevated (Fig 4). These results indicate that *P*. *densiflora* seedlings produce Fe detoxicants to adapt to heavy metal environments, and *M*. *sinensis* would protect *P*. *densiflora* seedlings from high soil temperature stress.

Root endophytes, AM mycorrhiza, and ectomycorrhiza increase heavy metal tolerance in plants [25–27,29,63–65]. Ectomycorrhiza can infect *P*. *densiflora* and enhance nutrient uptake [66]. However, under heavy metal stress, infection is inhibited, and root endophytes can infect plants instead of ectomycorrhiza. In a volcanic desert, *Salix reinii* infected with ectomycorrhiza can grow on patches formed by *Reynoutria japonica* [67]. However, in the present study, *P*. *densiflora* seedlings were not infected by ectomycorrhizal fungi but by root endophytes (Fig 6). The growth of ectomycorrhiza is inhibited by high concentrations of heavy metals in the soil to suppress infection in plants [68]. Therefore, root endophytes could contribute to Fe tolerance in *P*. *densiflora* seedlings in heavy metal environments. There were no differences between the root endophyte species isolated from *P*. *densiflora* seedlings outside and inside the patches, and *C*. *bicorne* and *Aquapteridospora* sp. were isolated at a high frequency. The root endophytic *Ceratobasidium* sp. enhances the growth of *Malus domestica* via increasing nutrient uptake [69]. As there are no reports on the enhancement of heavy metal tolerance by *Ceratobasidium* sp., inoculation tests using *C*. *bicorne* and *P*. *densiflora* seedlings under heavy metal stress should be conducted to clarify the contribution of *C*. *bicorne* to Fe tolerance in *P*. *densiflora* seedlings. *Aquapteridospora* sp. was discovered in 2015 and was classified as a new genus [70,71]. *Aquapteridospora* sp. had melanized dark hyphae, meaning that *Aquapteridospora* sp. could be a DSE. Several DSEs increased the growth and tolerance of the host plants, *Medicago sativa* and *Ammopiptanthus mongolicus*, under cadmium stress [72]. However, the effects of *Aquapteridospora* sp. on heavy metal tolerances in plants remain unclear. In this study, *Aquapteridospora* sp. was isolated from the roots of *M*. *sinensis* and *P*. *densiflora* seedlings inside the patches (Fig 7), suggesting that *M*. *sinensis* might provide *Aquapteridospora* sp. to *P*. *densiflora* seedlings. Therefore, *Aquapteridospora* sp. is an interesting fungus with ecophysiological functions. Inoculation tests on *P*. *densiflora* seedlings under heavy metal stress should be conducted to determine the function of *Aquapteridospora* sp. at the study site.

Colonization in plant tissues by root endophytes without symptoms is established by balanced antagonism between the host and endophyte; therefore, environmental stress and plant conditions could change the interactions between plants and root endophytes from symbiotic to pathogenic or saprophytic [73]. A previous report [74] classified endophytes into four categories below; Class 1 endophytes are Clavicipitaceous, Class 2 endophytes could show weakly pathogenic and saprophytic functions, Class 3 endophytes could infect only stems and leaves without diseases, Class 4 endophytes with dark septate hyphae could infect roots without pathogenic functions. For example, DSEs classified into Class 4 could increase heavy metal tolerance in plants [27,75] and saprophytically decompose dead plant tissues [76]. Because Class 4 endophytes do not show pathogenic functions [74], DSEs including *Aquapteridospora* sp., isolated from dead *P*. *densiflora* seedling roots (S1 Table) would grow saprophytically after *P*. *densiflora* seedlings died rather than as a mortality factor. *Ceratobasidium bicorne* isolated from *P*. *densiflora* seedlings outside patches at a high frequency (Fig 7) is classified as a Class 2 endophyte, suggesting that *C*. *bicorne* might exhibit pathogenic functions according to *P*. *densiflora* seedling conditions. Outside the patches, excessive increases and sharp changes in soil temperature were observed (Table 2; S2 Fig). The severe environment might weaken *P*. *densiflora* seedlings outside patches via suppressing defense systems [55,58], to change the interaction with *C*. *bicorne*, which might be not endophytic but weakly pathogenic. These results suggest that *M*. *sinensis* patch would suppress the sharp increase and alteration in soil temperature, which seem to be main stress for *P*. *densiflora* seedlings to survive under the sedimentary site. Previously, *M*. *sinensis* is thought to suppress the establishment of tree seedlings by competition of nutrient uptake and making shade [77]. However, our study indicated that *M*. *sinensis* would provide an appropriate environment via controlling soil temperature. As the results, *M*. *sinensis* could promote vegetation succession by *P*. *densiflora* establishment. In the future, inoculation tests using sterilized *P*. *densiflora* seedlings with *C*. *bicorne*, *Aquapteridospora* sp., or a mixture of both root endophytes under high soil temperature and heavy metal stresses should be conducted to clarify the contribution of *C*. *bicorne* and *Aquapteridospora* sp. to the Fe tolerance of *P*. *densiflora* seedlings. From the inoculation test, measurement of Fe concentration and detoxification and mortality rate in *P*. *densiflora* seedlings can be used to understand the contribution of both root endophytes to the establishment of *P*. *densiflora* at our study site.

## Conclusion

We studied the mechanisms by which *M*. *sinensis* could facilitate the survival of *P*. *densiflora* seedlings in the sedimentary site, which exposes plants to high soil temperature and Fe stress. *Pinus densiflora* seedlings produce Fe detoxicants to adapt to Fe stress. In contrast, high soil temperature would weaken *P*. *densiflora* seedlings and root endophytic *C*. *bicorne* might cause weak pathogenicity to reduce *P*. *densiflora* establishment in our study site. Up to the present, *M*. *sinensis* is thought to suppress the establishment of tree seedlings. However, in our study site, *M*. *sinensis* might promote the establishment of *P*. *densiflora* seedlings by suppressing environmental stresses and providing a DSE, *Aquapteridospora* sp, which would allow *P*. *densiflora* to adapt in severe environments, resulting in vegetation succession.

## Supporting information

S1 Fig Mortality factors of *Pinus densiflora* seedlings outside and inside patches. Percentage of mortality factors in *P*. *densiflora* seedlings collected from July 2020 to September 2022. Numbers above each bar indicate the number of dead seedlings.
(PDF)

S2 Fig Soil temperature outside and inside the patches from July 2022 to September 2022. (a) Soil temperature outside the patches and (b) soil temperature inside the patches. Soil temperatures were measured at a depth of 5 cm outside and inside the two patches.
(PDF)

S1 Table Detection rates of fungi isolated from dead *Pinus densiflora* seedlings with symptoms in July 2020. The number of dead *P*. *densiflora* seedlings collected from outside and inside the patches in July 2020, showing symptoms and detection rates of fungi isolated from dead *P*. *densiflora* roots.
(PDF)

S2 Table Comparison of harmful metal concentrations in roots of *Pinus densiflora* seedlings. DW; dry weight. Different letters indicate statistically significant differences among treatments in one-factor ANOVA, Scheffé post-hoc test, P < 0.05 (n = 5). The concentrations of heavy metals in the roots of each *P*. *densiflora* seedling were analyzed statistically. Results are expressed as mean ± standard error (SE).
(PDF)
