# Supplementary material for: Miscanthus sinensis contributes to the survival of Pinus densiflora seedlings at a mining site via providing a possible functional endophyte and maintaining symbiotic relationship between P. densiflora and endophytes from high soil temperature stress
Source: PLoS One. 2023 May 23;18(5):e0286203. doi: 10.1371/journal.pone.0286203 (PMC10204988; doi:10.1371/journal.pone.0286203)
Supplement: S1 Fig — Percentage of mortality factors in P. densiflora seedlings collected from July 2020 to September 2022. Numbers above each bar indicate the number of dead seedlings. (PDF) [file pone.0286203.s001.pdf]

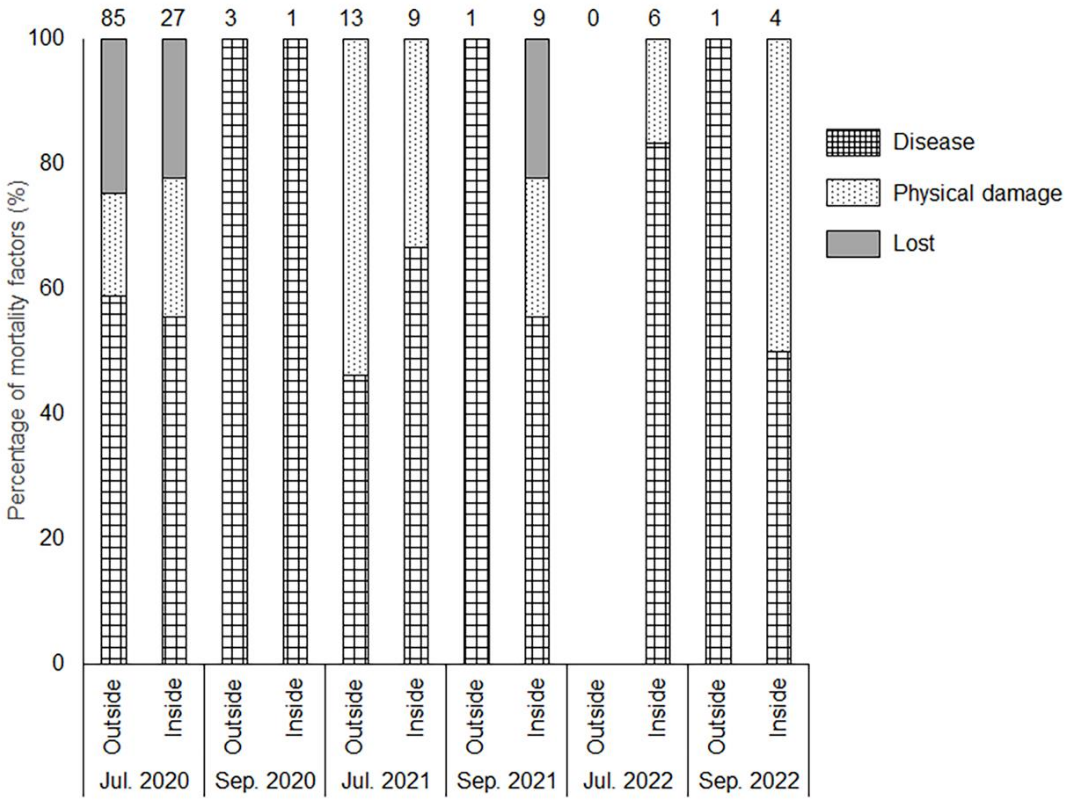

S1 Figure. Mortality factors of *Pinus densiflora* seedlings outside and inside patches.

Percentage of mortality factors in *P. densiflora* seedlings collected from July 2020 to September 2022. Numbers above each bar indicate the number of dead seedlings.
