# Supplementary material for: Miscanthus sinensis contributes to the survival of Pinus densiflora seedlings at a mining site via providing a possible functional endophyte and maintaining symbiotic relationship between P. densiflora and endophytes from high soil temperature stress
Source: PLoS One. 2023 May 23;18(5):e0286203. doi: 10.1371/journal.pone.0286203 (PMC10204988; doi:10.1371/journal.pone.0286203)
Supplement: S2 Fig — (a) Soil temperature outside the patches and (b) soil temperature inside the patches. Soil temperatures were measured at a depth of 5 cm outside and inside the two patches. (PDF) [file pone.0286203.s002.pdf]

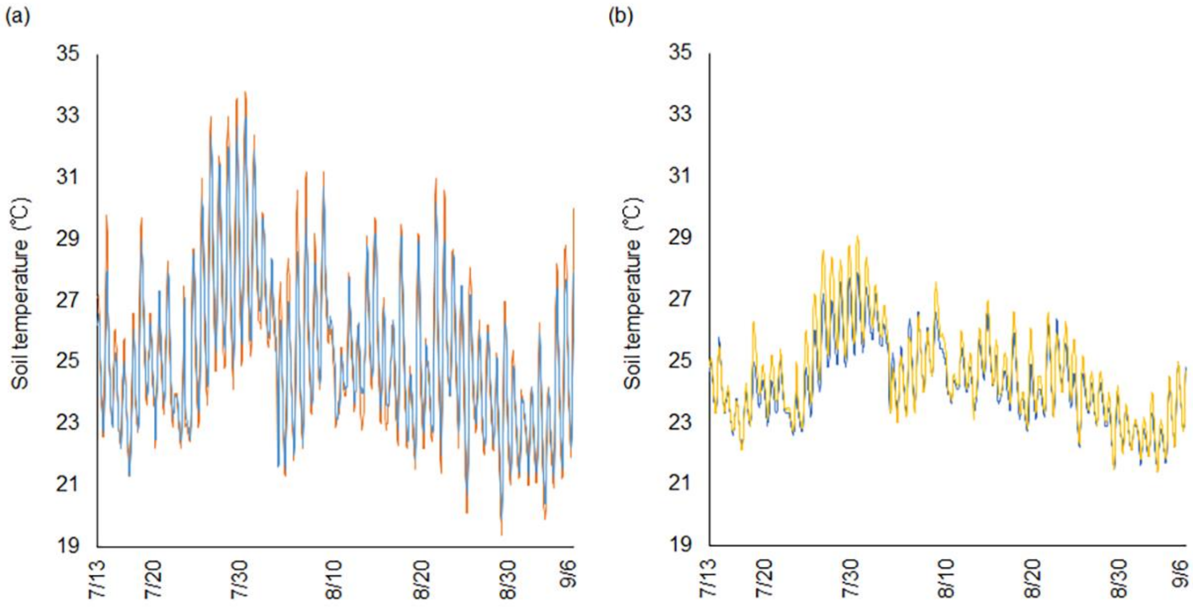

S2 Figure. Soil temperature outside and inside the patches from July 2022 to September 2022.

(a) Soil temperature outside the patches and (b) soil temperature inside the patches. Soil temperatures were measured at a depth of 5 cm outside and inside the two patches.
