# Supplementary material for: Miscanthus sinensis contributes to the survival of Pinus densiflora seedlings at a mining site via providing a possible functional endophyte and maintaining symbiotic relationship between P. densiflora and endophytes from high soil temperature stress
Source: PLoS One. 2023 May 23;18(5):e0286203. doi: 10.1371/journal.pone.0286203 (PMC10204988; doi:10.1371/journal.pone.0286203)
Supplement: S1 Table — The number of dead P. densiflora seedlings collected from outside and inside the patches in July 2020, showing symptoms and detection rates of fungi isolated from dead P. densiflora roots. (PDF) [file pone.0286203.s003.pdf]

S1 Table. Detection rates of fungi isolated from dead *Pinus densiflora* seedlings with symptoms in July 2020.

	No. of dead seedlings with symptoms	Detection rate of *C. bicorne* (%)	Detection rate of DSEs (%)
Outside	50	12.0	74.0
Inside	15	0	100

The number of dead *P. densiflora* seedlings collected from outside and inside the patches in July 2020, showing symptoms and detection rates of fungi isolated from dead *P. densiflora* roots.
