# Supplementary material for: Miscanthus sinensis contributes to the survival of Pinus densiflora seedlings at a mining site via providing a possible functional endophyte and maintaining symbiotic relationship between P. densiflora and endophytes from high soil temperature stress
Source: PLoS One. 2023 May 23;18(5):e0286203. doi: 10.1371/journal.pone.0286203 (PMC10204988; doi:10.1371/journal.pone.0286203)
Supplement: S2 Table — DW; dry weight. Different letters indicate statistically significant differences among treatments in one-factor ANOVA, Scheffé post-hoc test, P < 0.05 (n = 5). The concentrations of heavy metals in the roots of each P. densiflora seedling were analyzed statistically. Results are expressed as mean ± standard error (SE). (PDF) [file pone.0286203.s004.pdf]

S2 Table. Comparison of harmful metal concentrations in roots of *Pinus densiflora* seedlings.

Element (mg/kg DW)	Jul. 2020		Sep. 2020		Jul. 2021		Sep. 2021	
	Outside	Inside	Outside	Inside	Outside	Inside	Outside	Inside
Al	4486.7 ± 275.5b	4556.7 ± 743.5b	2893.4 ± 210.6b	2634.7 ± 228.2b	6085.9 ± 531.7c	4798.0 ± 930.1bc	4565.2 ± 601.5b	3826.1 ± 395.9b
Cu	1121.7 ± 82.9a	1389.0 ± 218.6ab	868.4 ± 87.3a	959.2 ± 83.1a	2718.7 ± 442.7b	2228.9 ± 382.3ab	1645.5 ± 195.8a	2014.4 ± 184.9ab
Fe	8002.7 ± 580.5c	9157.4 ± 1764.4c	5065.4 ± 505.1c	5752.5 ± 502.8c	7842.5 ± 963.8c	8143.8 ± 1667.5c	7302.8 ± 1061.3c	6504.7 ± 1108.4c
Mn	99.6 ± 9.4a	86.5 ± 10.8a	100.6 ± 16.2a	70.9 ± 8.8a	145.3 ± 12.3a	157.5 ± 38.8a	121.0 ± 16.3a	116.0 ± 19.3a
Zn	1302.9 ± 89.3a	1712.4 ± 197.2ab	900.2 ± 78.5a	1175.7 ± 154.5a	1914.9 ± 244.6ab	2009.8 ± 317.4ab	1435.9 ± 180.7a	1770.0 ± 161.7ab

DW; dry weight. Different letters indicate statistically significant differences among treatments in one-factor ANOVA, Scheffé post-hoc test, $P < 0.05$ ($n = 5$). The concentrations of heavy metals in the roots of each *P. densiflora* seedling were analyzed statistically. Results are expressed as mean ± standard error (SE).
